# Supplementary material for: A qualitative photo-elicitation study exploring the impact of falls and fall risk on individuals with subacute spinal cord injury
Source: PLoS One. 2022 Jun 7;17(6):e0269660. doi: 10.1371/journal.pone.0269660 (PMC9173606; doi:10.1371/journal.pone.0269660)
Supplement: S1 Checklist — Completed COREQ (Consolidated criteria for reporting qualitative research) checklist. (DOCX) [file pone.0269660.s001.docx]

**COREQ Checklist**

|  | Included |
| --- | --- |
| **Domain 1: Research team and reflexivity** | |
| Personal characteristics |  |
| 1. Interviewer/facilitator | Page 7 |
| 1. Credentials | Pages 7,9 |
| 1. Occupation | Pages 7,9 |
| 1. Gender | Page 8 |
| 1. Experience and training | Pages 7,9 |
| Relationship with participants |  |
| 1. Relationship established | Page 7 |
| 1. Participant knowledge of the interviewer | Page 7 |
| 1. Interviewer characteristics | Page 7 |
| **Domain 2: Study Design** | |
| Theoretical framework |  |
| 1. Methodological orientation and Theory | Page 5,6 |
| Participant selection |  |
| 1. Sampling | Page 6 |
| 1. Method of approach | Page 6 |
| 1. Sample size | Page 6,7 |
| 1. Non-participation | Page 9 |
| Setting |  |
| 1. Setting of data collection | Page 7 |
| 1. Presence of non-participants | Not applicable |
| 1. Description of sample | Page 9-10 |
| Data collection |  |
| 1. Interview guide | Page 8, Supplementary file |
| 1. Repeat interviews | Not applicable |
| 1. Audio/visual recording | Page 8 |
| 1. Field notes | Not applicable |
| 1. Duration | Page 7 |
| 1. Data saturation | Page 31 |
| 1. Transcripts returned | Not conducted |
| **Domain 3: Analysis and findings** | |
| Data analysis |  |
| 1. Number of data coders | Page 8 |
| 1. Description of the coding tree | Page 8-9 |
| 1. Derivation of themes | Page 8-9, 10-26 |
| 1. Software | Not applicable |
| 1. Participant checking | Not conducted |
| Reporting |  |
| 1. Quotations presented | Page 10-26 |
| 1. Data and findings consistent | Page 10-26 |
| 1. Clarity of major themes | Page 10-26 |
| 1. Clarity of minor themes | Page 10-26 |
