# Supplementary material for: A qualitative photo-elicitation study exploring the impact of falls and fall risk on individuals with subacute spinal cord injury
Source: PLoS One. 2022 Jun 7;17(6):e0269660. doi: 10.1371/journal.pone.0269660 (PMC9173606; doi:10.1371/journal.pone.0269660)
Supplement: S1 File — Semi-structured interview guide. (DOCX) [file pone.0269660.s002.docx]

**Interview Guide**

**1**. Confirm whether participant has chosen 2-3 pictures for each of these questions:

1. What increases your likelihood of falling?
2. What decreases your likelihood of falling?
3. How does the risk of falling affect your ability to participate in work (paid or volunteer)?
4. How does the risk of falling affect your ability to participate in recreational activities?

**2**. SHOWeD Framework [1]

For each picture that the participant would like to discuss, ask the following:

a) What do you **S**ee here?

b) What is really **H**appening here? [INTERVIEWER PROBE: What does the picture represent to you? What is happening that caused you to take this photo?]

c) How does this relate to **O**ur lives? [INTERVIEWER PROBE: How does this issue or factor impact your life?]

d) **W**hy does this situation, concern or strength **e**xist?

e) What can we **D**o about it? [[INTERVIEWER PROBE: How do you think the situation could be improved?]

**3**. Open-ended Questions

Falls are quite common in ambulatory and wheelchair users with SCI. The focus of this interview is to learn about your perceptions about falls and fear of falling and how you can be better prepared for falls while in rehabilitation.

1. How do you define a fall?
2. How many falls have you had since sustaining your spinal cord injury? Tell me about what caused the fall.

PROBE: How did it affect you (i.e. restrict certain activities)? How did you get up from the fall?

1. Do you have a fear of falling? What strategies do you use to prevent falls? How did you develop those strategies?

PROBE: What are three of your most useful falls prevention strategies?

1. How would you define falls training?

PROBE: What would an ideal falls training program include?

1. Could you please describe in detail any advice or training on falls you received while in the rehabilitation hospital?

PROBES: How helpful was this advice? Who provided this advice? How much of a focus were falls in rehabilitation?

Are there any other sources you use for advice on preventing falls?

In your opinion, was the falls training you received from the rehabilitation hospital adequate?

How satisfied are you with the level of falls training you received from rehabilitation?

1. In what ways could falls training in rehabilitation hospitals be made more useful for you?

PROBE: Effectiveness, program delivery, learning materials (handouts)?

1. In your opinion, what should the focus of falls prevention training be in rehabilitation?

PROBE: How to prevent a fall or how to fall safely?

1. Based on your experience in rehabilitation, who would be the most appropriate person to provide falls training? Why would this be the most appropriate person?
2. Do you think hospital staff and people with SCI have different views about falls? Please explain.
3. In your opinion, when is the best time to provide falls training? Please explain.

PROBE: Why would it be better than training at this time?

1. Please suggest at least 3 specific ways rehabilitation could prepare you better for falls (e.g. more focus on falls education, falls training, etc.)
2. Is there anything else you would like to discuss about falls and fall prevention?

[1] Wang CC. Photovoice: a participatory action research strategy applied to women’s health. J Women's Health. 1999;8(2):185-192.
