# Supplementary material for: A qualitative photo-elicitation study exploring the impact of falls and fall risk on individuals with subacute spinal cord injury
Source: PLoS One. 2022 Jun 7;17(6):e0269660. doi: 10.1371/journal.pone.0269660 (PMC9173606; doi:10.1371/journal.pone.0269660)
Supplement: Attachment — Submitted filename: Response to Reviewers.docx [file pone.0269660.s003.docx]

Thank you to the Reviewers and Editor for the helpful comments on our manuscript. We have addressed all of the comments through revisions to the manuscript, which are outlined below. Reviewers’ comments are in italics. Text that we added to the revised manuscript is underlined. The page and line numbers refer to the tracked version of the revised manuscript.

Comments from the Editor:

*Specifically, issues related to qualitative methodology (e.g., coding scheme, saturation, sampling), and how any methodological choices may have influenced study results (e.g., older sample may suggest aging related changes in fall risk) were highlighted by both reviewers.*

Thank you for highlighting the critical points. We have addresses these methodological issues in our responses to the Reviewers below.

Comments from Reviewer 1:

1. *Abstract- The abstract is well written summarizing key points of the manuscript.
   Minor comments- Line 27 Instead of “inpatients with a new SCI/D…” consider “Individuals with SCI undergoing inpatient rehabilitation due to a new SCI/D…”*

We have made this edit. The sentence in the abstract now reads: Eight individuals (7 male) undergoing inpatient rehabilitation at a Canadian tertiary rehabilitation hospital due to a new SCI/D participated.

We have also shortened the Abstract to adhere to the word limit of 300 words.

2. *Introduction: The authors present a good rationale for investigating falls in the subacute population.
   Line 68 – The authors state “only 7-13%...” The use of the word only implies bias that this percentage is not of concern. This number is still of concern and relevant to falls in the hospital setting. Suggest stating the percent of those in inpatient that fall and contrast that with a percent of individuals who fall after discharge so the comparison is seen more factually without the introduction of bias or minimizing the problem of falls in the inpatient setting.*

This is a good point. We have made the following edits to this sentence in the Introduction (lines 73-76): It is estimated that 7-20% of rehabilitation inpatients with SCI/D fall [14-16], while 69-78% of individuals living with chronic SCI/D fall each year [1]; hence, a higher fall incidence likely manifests after individuals with SCI/D have been discharged from inpatient hospital care.

We also added a relevant paper to our reference list: Wilson A, Kurban D, Noonan VK, Krassioukov A. Falls during inpatient rehabilitation in spinal cord injury, acquired brain injury, and neurologmusculoskeletal disease programs. Spinal Cord. 2020 Mar;58(3):334–40.

3. *Methods- Overall, the methods are described clearly.
   Line 104, similar recommendation to what was said in the abstract on line 27*

We have edited this sentence as follows (lines 110-111): Individuals undergoing inpatient rehabilitation at the Lyndhurst Centre were invited to participate through a centralized recruitment process [26].

4. *Methods - Line 114- The authors should provide additional information on why 7-10 was the target sample size. It says this was based on previous research - but what about the previous research led the researchers to arrive at this number? Was this enough to reach saturation of data for example? The COREQ checklist says saturation is addressed on p6-7 but this is the only line that addresses sample and saturation is not addressed directly.*

A sample size of 7-10 participants is recommended in the original photovoice research papers as this group size “allow[s] for practical ease and in-depth discussion” (Wang 2006). We have added this information to the Methods (lines 121-125): Our desired sample size of 7-10 individuals was based on previous photo-elicitation research [11, 28, 29] and the sample size recommended for photo-based group discussion [30]. Participants of the current study engaged in a focus group meeting as part of the larger research project, and a sample size of 7-10 participants enabled in-depth group discussion [30].

We have also added discussion about the small sample size and data saturation in the limitations paragraph of the Discussion (lines 622-629): Third, although our sample size of eight participants was within the recommended range for photo-elicitation research [11, 28-30], this sample size is small. Data saturation, or the point when no changes or additions to the codebook result from additional interviews [51], may not have been reached in our study. However, prior research that also involved a nonprobabilistic, purposive sample demonstrated that 80% of the codes were identified after the first six transcripts and 92% of the codes after 12 transcripts [51]. Hence it is reasonable to assume that additional interviews would not have led to significant changes to the codebook.

In addition, the COREQ checklist has been updated and uploaded as a supplemental file.

5. *Methods - Line 131-133 – It is great that the authors offered this modification though this does not seem to add anything to the manuscript*

We have deleted these lines in the revised manuscript.

6. *Methods - Line 153-155- recommend describing codes in the authors own words as it relates to this topic and citing reference 31 vs the direct quote as this may be more meaningful to the reader*

We have removed the quote from this sentence and instead state (lines 163-166): Codes represent basic elements or pieces of information about the studied phenomenon that are identified in the transcribed dialogue [33].

7. *Methods - Line 153-155 Authors generate ideas for codes and then SM applied the coding scheme. What happened between this step? How was the coding scheme agreed upon?*

We have provided additional detail about the coding process (lines 162-169): The same researchers then discussed as a group and interpreted the data to generate initial ideas for codes. Codes represent basic elements or pieces of information about the studied phenomenon that are identified in the transcribed dialogue [33]. Through discussion, the four researchers reached agreement on an initial codebook (i.e. code labels with definitions). SM then applied this coding scheme to each transcript manually, discussing any edits or additions to the codebook as they arose with the other three researchers.

8. *Methods - Line 157- Why is theme 4 the only one that has subcategories? Could the methods provide more information on the division of themes to categories to subcategories and how that was done?*

We have added the following sentences to the Methods to address this comment (lines 170-173): Codes that represented similar concepts were grouped together to form subcategories or categories, depending on the number of levels of hierarchy that emerged in the data. Themes were identified for categories that grouped together in a meaningful way.

9. *Results-
   171- The reader is left wondering why only 8 individuals participated when 45 were invited?*

Yes, we were surprised by this low recruitment rate as well. We suspect the stage of recovery may have been a factor, as well as the passive recruitment method used. We have added more information about how participants were recruited in the Methods (lines 117-121): From the group of 60 participants that were recruited for the larger study, participants who lived within 100km of the Lyndhurst Centre were invited to participate in photo-elicitation approximately six months after discharge from inpatient rehabilitation. These individuals were sent a recruitment flyer through email or the postal service.

We also added a few sentences to the paragraph on limitations in the Discussion (lines 629-634): Fourth, we were surprised to recruit only eight participants from the 45 individuals sent the recruitment flyer. We did not systematically track the reasons why participants chose not to participate, as potential participants contacted the research team only if they were interested in participating in the photo-elicitation study. However, a few participants volunteered reasons why they chose not to participate, which included being too busy with community-based rehabilitation or other medical appointments, and preparing for a return to work.

10. *Results - Lines 174 and Table 1 – The authors clearly establish that all participants were ambulatory except 1. This is clear and it seems unnecessary and distracting to then note “ambulator” after each participant code throughout the manuscript. The authors could indicate F56 as a manual wheelchair user if they want to distinguish, but since the reader already knows the rest are ambulatory, this feels redundant and distracting.*

We have removed “ambulator” after the participant codes in the manuscript and the figures.

11. *Results - Line 189 and Table 2 – Nice summary of themes and supporting quotes to support the categorization. Category 2a only has 1 quote and 4b has no quotes. For consistency it seems each should have at least 2 quotes to show support for the categories.*

We have added quotes to Table 2, such that category 2a, and the subcategories in 4b have at least two example quotes.

12. *Results - The authors summarize each theme, category, and subcategory in the remainder of the results providing support from the interviews for each. It is unclear why some quotes are embedded directly in the paragraphs and some quotes are presented in italics. What is the difference in how these are presented and why?*

We have followed APA guidelines for presenting quotes in manuscripts (i.e. if <40 words, use quotations to embed within the sentence). We have edited a few quotes in the revised manuscript that were formatted incorrectly in the previous version.

13. *Discussion - Line 490 – Authors need to make clear that the recommendations came from the participant data*

We have added the following words to the Discussion to clarify this point (lines 530-535): A synthesis of the findings resulted in recommendations for fall prevention education and training throughout the first year after SCI/D (see Table 3). These recommendations, which were derived from the participants’ data and prior literature, are applicable to and desired by clinicians and rehabilitation programs [18, 19] involved in facilitating the transition to community living after SCI/D.

14. *Discussion - Lines 498-509 The authors address the issue that all of the participants had non-traumatic SCI and that half were over age 65. Did the authors accomplish their goal with this participant pool as the results could reflect SCI and age related fall risk factors? Results may have been different if the population was more representative of the demographics of SCI. The authors state in line 498 that “it is noteworthy”. This is more than just noteworthy and is a major limitation of the manuscript and should be addressed in limitations. Would the themes have come out the same way if younger individuals with traumatic injuries were included in the participant pool? The paper still presents good qualitative data for the 8 individuals with non traumatic SCI though this is a big limitation.*

We appreciate the reviewer’s point. We have added a statement to the limitations paragraph in the Discussion (see below); however, the characteristics of our eight participants do not differ drastically from institutional- and national-level data, and we have attempted to strengthen this point in the revised manuscript. At the rehabilitation hospital where this research was conducted, roughly 70% of inpatient admissions over the past two years have been non-traumatic injuries (unpublished data). A publication from ten years ago stated that there were twice as many non-traumatic SCI/D inpatient admissions than traumatic admissions at Canadian hospitals. The greater proportion of non-traumatic injuries likely explains the high mean ages of individuals with SCI/D who are receiving inpatient rehabilitation in Canada (i.e. >50 years of age). These points have been added to the Discussion, along with a statement of limitation:

Lines 540-556: All eight participants in this study experienced non-traumatic damage to their spinal cord. To date the majority of the research on falls in this population has focused on those with traumatic causes of injury [2, 3, 5, 8-10, 12, 13, 37]; however, the demographics of SCI/D are changing [38-41]. The incidence of non-traumatic causes of SCI/D is increasing, with the prevalence now estimated to exceed that of traumatic injury in some countries [42, 43]. In Canada, it is estimated that there are two times as many non-traumatic cases of SCI/D each year compared to traumatic injuries. [41] Consistent with the increase in non-traumatic SCI/D, the age of onset of SCI/D is increasing and more individuals are presenting with pre-existing health conditions [38, 41]. Both increasing age and co-morbid conditions are known risk factors for falls [44]. All but one of our participants were aged ≥50 years, and half of the participants were aged 65 years or greater. However, these ages are typical for SCI/D rehabilitation in Canada. The majority of those with SCI/D receiving inpatient rehabilitation in Canadian hospitals are >50 years of age [41], with the mean age of those receiving inpatient rehabilitation due to non-traumatic injuries being 59.0 ± 18.5 years. [45] The issues and concerns described in this study may reflect both aging and sustaining non-traumatic SCI/D, and may have differed if our participants were primarily younger individuals with SCI/D or those with traumatic injuries.

Lines 634-636: Fifth, all eight participants had experienced non-traumatic SCI/D; hence, the perspectives of those with subacute, traumatic injuries are not reflected in the study findings.

We agree that age may have influenced perceptions of falls and fall risk (as stated in the Discussion); however, age-related concerns were not commonly mentioned by our participants. Participant F56 was one exception; they did express concern about aging. We have added a quote to Table 2 (final quote in Table 2).

15. *Discussion - Bottom p28-top p29- Another concept that would be worth discussing related to the fall prevention training is individual’s readiness to learn. As stated in the limitations, PTs and OTs may include fall education and authors state that they may not label it as fall prevention training, but also patients may not be ready to hear or absorb the information at that time or think that falls would ever even be an issue given their perception of recovery and readiness to learn. The patient perception of it being included in their rehabilitation is important, regardless of if it was or was not, but acknowledging a possibility that it was included and not absorbed at the time is important.*

We agree that readiness to learn is an important factor to consider. We have added the following sentences to the Discussion (lines 606-610): Moreover, individuals with SCI/D have previously described hospitalization after SCI/D as a time of distress and confusion, and were unprepared to receive education [49]. Several of our study participants shared similar sentiments; inpatient rehabilitation was a time when they felt overwhelmed and did not appreciate the relevance of fall prevention education and training.

16. *Conclusion: Suggest revising the conclusion to directly connect to the themes as the conclusion does not fully support the data that is presented.
    The conclusion in the discussion states that the study describes the impact of falls and fall risk. In the abstract the conclusion states that falls can have a significant impact on the first year of living… These are different and should be consistent, though see above suggestion for revision. ( It would also be helpful to know how many falls did occur during that 6 month period. Was this asked of the participants? While this is not qualitative data, it would help give some context) . One of the quotes specifically states the participant has not fallen, but beyond that the reader does not know.)*

We have added the number of falls that each participant experienced in the first six months post-discharge from inpatient rehabilitation in Table 1.

We have revised the Conclusion as follows (lines 639-646): In conclusion, the study findings have described the impact of falls and fall risk during the transition from hospital to community living from the perspectives of eight individuals with non-traumatic SCI/D. Participants discovered their fall risk factors and fall prevention strategies through lived experience with falls, which included falls experienced by themselves as well as friends and family. A high fall risk caused participants to alter participation in daily activities, increased negative emotions and reduced independence and quality of life. Participants’ experiences with falls and fall prevention varied; however, all agreed that comprehensive and individualized fall prevention education and training was needed.

The Conclusion in the Abstract has been revised as follows (lines 50-51): Although participants’ experiences with falls and fall prevention varied, falls and the risk of falling can have a significant impact on the first year of living with a SCI/D.

17. *The conclusion also states that findings may inform future fall prevention initiatives. This is very generalizable. It may be more appropriate to state conclusions related to the themes and what was found with these 8 individuals. The variability of responses related to experiences and opinions about fall prevention appears to be a more clear conclusion from the data.*

Thank you for these suggestions, which we considered when we revised the conclusions in the Abstract and at the end of the manuscript. These changes are outlined above, in response to comment 16.

Comments from Reviewer 2:

1. *Since the current demographics show that half of the total participants were above 65 years of age, did this subgroup of sample have a previous fall history or fear of falling associated with aging related changes even before the SCI/D? Do the authors consider that these individuals are more likely to restrict their participation in walking activities (due to aging related balance deficits, fear of falling) and thus might encounter fewer circumstances that might increase potential fall-risk?*

We do not know the participants’ pre-SCI/D fall history or fear of falling status; however, we agree that aging may have an influence on participants’ perceptions of their fall risk and participation restrictions. Please see our response to Reviewer #1’s comment 14.

2. *Given the heterogenous sample including varying level of neurological involvement, could the author comment about the influence of perceived fall-risk for individuals with cervical vs lumbar levels. Also, did the authors come across any interaction between behavioral factors and level of involvement. For eg. Individuals with cervical level of involvement showed lower risk-taking behavior and are more cautious of their surroundings resulting in identification of more risk-factors and use of strategies to prevent falls, compared to those with lumbar level who might demonstrate greater risk-taking behavior.*

This is an interesting idea. Differences between those with living paraplegia versus tetraplegia did not emerge from the data; however, as we only had 3 individuals living with paraplegia and five individuals with tetraplegia, a sub-analysis was not completed. A 2019 review article (Khan et al. Spinal Cord; 2019: 57: 526) listed the factors currently known to contribute to falls after SCI. Severity of injury, but not level of neurological injury, has previously been identified (Table 3 in Khan et al.).

3. *Was there any sub-analysis performed to determine whether those who experienced falls during the 6 months after discharge vs non fallers demonstrated any difference in their perceived fall-risk or impact of falls in their activity participation?*

No sub-analysis comparing those who fell (n=4) and those who did not fall (n=4) during the study period was planned or performed. Based on our knowledge of the data, we do not think there were differences in the perceived fall-risk of these two small groups. Under Theme 2: Influences on the individual’s perception of their fall risk, it is discussed how participants referred to not only falls they experienced themselves since sustaining a SCI/D as influencing their perceived risk, but also falls experienced before their SCI/D and falls experienced by friends and family members. We thought this was an interesting finding as it differed from prior literature involving individuals living with chronic SCI/D (lines 560-566): However, in contrast to the chronic population, those in the subacute phase of SCI/D referenced learning through the experiences of friends and family in addition to their own experiences. This difference may reflect that those in the subacute phase of injury have had fewer exposures to falls, and thus, less experience to draw from. There are several implications of this finding. First, fall prevention education during the subacute phase may benefit from the inclusion of real-world examples of falls, perhaps from those living with chronic SCI/D…

4. *Additionally, during this period, did the participants undergo any balance and/or locomotor training or fall education intervention? Was this information collected and analyzed to understand the potential implications on change in fall-risk factors among those who received intervention vs those who did not?*

In Canada, the majority of individuals with SCI/D receive publicly funded outpatient therapy following their inpatient stay. Participants in this study were likely finishing or had recently finished their outpatient therapy, but we did not include questions about current balance/mobility/exercise training when collecting demographic information. We gained knowledge about their experiences with fall prevention training in the semi-structured interview (lines 152-154): Additional interview questions concerning their experiences and opinions about fall prevention training in rehabilitation were asked. See supplementary material for the interview guide.

These interview questions resulted in reflection on their own experiences, which are discussed in Theme 4: Falls training in rehabilitation can be improved.

5. *Do authors think that information regarding participants housing is necessary to be included as a contributor towards fall-risk, since inability to fall-proofing the house might lead to greater fall-risk?*

We agree that the inability to fall-proof one’s home could lead to greater fall risk. Participants did discuss how the environment affected their fall risk (i.e. Category 1b. The environment is often less accessible to individuals with SCI/D and increases fall risk); however, the majority of examples and photos that contributed to this category were focused on environments outside of their home. We did not collect information about participants’ living environment when we collected demographic information.

6. *Inclusion of uneven number of males and females might be a potential limitation of the study since gender specific disparities (if any) would be overlooked given that majority of participants were males.*

This is a good point. We have added the following comment to the limitations section of the Discussion (lines 636-638): Lastly, all but one participant identified as male (i.e. 87.7% of the sample). In Canada, approximately 57% individuals with non-traumatic SCI/D are male [45], meaning that males were over-represented in our sample.

7. *During the in-person interview, authors mention about non-verbal cues were given. What was the nature of these cues and could it possibly influence the results? If yes, how did the authors control for participants bias?*

We have edited the statement about non-verbal cues In the Discussion to improve clarity (lines 612-617): The current study has several limitations to note. First, the majority of interviews were conducted in person; however, three interviews were completed over the phone. In addition to lacking non-verbal communication (e.g. body language, hand gestures, gaze), the dialogue of phone interviews is known to differ from that of in person interviews [50]. For example, interviewees tend to ask for more clarification during phone interviews and phone interviews tend to be shorter in duration [50].

The absence of non-verbal communication in the phone interviews may have impacted the quality of the dialogue and that is why we have included this point as a limitation.

8. *Page 8 line 153-155: It would be helpful to be more specific regarding how the analysis was performed using the codes. Also, detailed explanation regarding ‘information that can be assessed in a meaningful way regarding the phenomenon’ will be valuable.*

We have provided greater detail about the analysis. Please see our responses to Reviewer #1’s comments 6, 7 and 8.

9. *Page 6 line 61: correct to ‘reduced work productivity’*

This change has been made (line 66).

10. *Page 27 line 536-537: “However, the participants with subacute SCI/D noted the challenge of influencing community environments.” This sentence is confusing and not very clear.*

We have edited this sentence to increase clarity (lines 581-586): Consistent with other studies [2, 11], the current participants mentioned making changes within their indoor living environment to reduce their fall risk and establish “an accessible proximal environment” [47]. However, the current participants with subacute SCI/D noted the challenge of changing community environments. In contrast, those living with chronic SCI/D have explained that over time they learned strategies to influence the people and communities around them, such as educating others about accessibility [47].

Additional Comments from the journal:

1. *Please ensure that your manuscript meets PLOS ONE's style requirements, including those for file naming. The PLOS ONE style templates can be found at
   https://journals.plos.org/plosone/s/file?id=wjVg/PLOSOne_formatting_sample_main_body.pdf and* 
   [*https://journals.plos.org/plosone/s/file?id=ba62/PLOSOne_formatting_sample_title_authors_affiliations.pdf*](https://journals.plos.org/plosone/s/file?id=ba62/PLOSOne_formatting_sample_title_authors_affiliations.pdf)

We have reviewed PLOS ONE’s study requirements and made any required changes to our manuscript, such as increasing the font of headings, shortening the abstract and embedding figure legends into the manuscript.

2. *Please update your submission to use the PLOS LaTeX template. The template and more information on our requirements for LaTeX submissions can be found at* [*http://journals.plos.org/plosone/s/latex*](http://journals.plos.org/plosone/s/latex)*.*

We did not use LaTeX for our submission.

3. *Your abstract cannot contain citations. Please only include citations in the body text of the manuscript, and ensure that they remain in ascending numerical order on first mention.*

We reviewed our abstract and cannot find any citations within.

4. *We note that Figures 2, 3 and 4 in your submission contain copyrighted images. All PLOS content is published under the Creative Commons Attribution License (CC BY 4.0), which means that the manuscript, images, and Supporting Information files will be freely available online, and any third party is permitted to access, download, copy, distribute, and use these materials in any way, even commercially, with proper attribution. For more information, see our copyright guidelines:* [*http://journals.plos.org/plosone/s/licenses-and-copyright*](http://journals.plos.org/plosone/s/licenses-and-copyright)*. We require you to either (1) present written permission from the copyright holder to publish these figures specifically under the CC BY 4.0 license, or (2) remove the figures from your submission:*

*Please upload the completed Content Permission Form or other proof of granted permissions as an "Other" file with your submission.*

*In the figure caption of the copyrighted figure, please include the following text: “Reprinted from [ref] under a CC BY license, with permission from [name of publisher], original copyright [original copyright year].”*

We were able to obtain completed Content Permission Forms from two of three participants for Figures 2, 3 and 4. The third participant verbally agreed to using their photos, but we had not received their signed form at the time of re-submission. This participant’s photos have been removed from Figures 2 and 4. If we receive the outstanding Content Permission Form, we will notify the journal. The complete Content Permission Forms have been uploaded as “Other” files with the revised manuscript.

*5. Please upload a copy of Supporting Information "Consolidated criteria for reporting qualitative research (COREQ):
754 32-item checklist" which you refer to in your text on page 39.*

The COREQ checklist was included with our original submission at the end of the manuscript document. We have uploaded it as a supplementary file for the revised submission. The checklist has been edited in the revised version to update the page numbers.

*6.* *Please review your reference list to ensure that it is complete and correct. If you have cited papers that have been retracted, please include the rationale for doing so in the manuscript text, or remove these references and replace them with relevant current references. Any changes to the reference list should be mentioned in the rebuttal letter that accompanies your revised manuscript. If you need to cite a retracted article, indicate the article’s retracted status in the References list and also include a citation and full reference for the retraction notice.*

No papers referenced have been retracted. One article that we referenced was previously under review. It has since been published and the full publication details have been added to the revised manuscript.
